# Development of an Immunoassay Platform Targeting β-1,3- and β-1,6-Glucans for Rapid Detection of Fungi

**DOI:** 10.3390/jof12060448

**Published:** 2026-06-19

**Authors:** Wei Yuan, Zan Chen, Yingyin Gao, Changbin Jin, Zhibo Yang, Wenzhuang Zhu, Di Zhang, Yueping Zhang

**Affiliations:** 1 State Key Laboratory of Veterinary Public Health and Safety, College of Veterinary Medicine, China Agricultural University, Beijing 100193, China; 2 China Agricultural University Veterinary Teaching Hospital, Beijing 100193, China; 3 Teaching Animal Hospital, China Agricultural University, Beijing 100193, China

**Keywords:** β-1,3-D-glucan, β-1,6-D-glucan, sandwich ELISA, fungal diagnostics

## Abstract

Fungal infections pose diagnostic challenges in both human and veterinary medicine, as traditional detection methods such as fungal culture are time-consuming, microscopy is operator-dependent, and molecular detection assays often require specialized instrumentation and trained personnel, which can limit their routine clinical application. This study developed a sandwich immunoassay to detect β-1,3- and β-1,6-glucans, two major components of the fungal cell wall, based on two catalytically inactive glucanase mutants, LamA_E175Q_ and Neg1_E321Q_. The sandwich ELISA exhibited higher detection sensitivity than conventional ITS-based PCR for *Saccharomyces cerevisiae* and *Candida albicans* under the conditions of this study. Using pre-coated plates, the sample-processing and detection workflow can be completed in approximately 40 min. It effectively detected a wide range of fungal species, including yeasts (*Saccharomyces cerevisiae*, *Candida albicans*) and filamentous fungi such as dermatophytes and non-dermatophyte molds. In a preliminary clinical cohort, the assay identified β-glucan signals in all 21 samples confirmed positive for dermatophytes, while no signal was detected in 20 negative samples, suggesting potential clinical applicability. This dual-enzyme sandwich immunoassay provides a rapid and low-cost complementary tool for broad-spectrum fungal screening, which may help guide further confirmatory diagnostics and timely clinical decision-making.

## 1. Introduction

Fungal infections are an increasing global health concern, affecting both humans and animals, with significant clinical, economic, and public health implications. In human medicine, opportunistic systemic mycoses caused by *Candida*, *Aspergillus*, and *Cryptococcus* species are leading causes of morbidity and mortality among fungal infections, particularly in immunocompromised patients [1,2]. In veterinary medicine, fungal diseases represent a significant health concern, encompassing both invasive mycoses and superficial dermatophytosis in a wide range of animal species. Invasive mycoses, which are more severe and systemic, include infections such as aspergillosis and candidiasis. These infections can affect multiple organs and often occur in immunocompromised animals, where they are associated with high morbidity and mortality [3]. On the other hand, dermatophytosis, caused by dermatophytes such as *Microsporum canis* and *Trichophyton mentagrophytes*, is a common and typically less severe fungal infection that affects a wide range of hosts, including humans, companion animals, and livestock, primarily involving the skin and hair. Although usually superficial, these infections can cause significant discomfort and are often zoonotic, posing a risk of transmission between animals and humans [4,5]. Dermatophytes are among the most prevalent fungal pathogens in animals, and zoonotic transmission represents an important route of spread, highlighting the need for reliable and efficient diagnostic methods [6,7]. *Alternaria*, a group of dematiaceous molds, is also a notable cause of fungal infections, leading to skin infections, respiratory issues, and allergic reactions in both humans and animals. These molds, while less commonly discussed than dermatophytes, pose significant threats in veterinary settings and are increasingly recognized as important pathogens [8,9].

Despite the critical nature of fungal infections, diagnostic approaches remain heavily reliant on traditional methods such as fungal culture and microscopy. While these methods have long been the standard, they are time-consuming, operator-dependent, and often limited by low sensitivity, particularly in cases of low-burden infections [5].

Molecular methods such as PCR are widely used for fungal detection and provide high analytical performance, particularly through the use of universal primers targeting the internal transcribed spacer (ITS) region [10]. However, despite their broad applicability [11], PCR-based assays generally rely on specialized laboratory infrastructure, including thermal cyclers, dedicated reagents, and trained personnel [12]. These requirements may limit their rapid deployment and routine implementation in some veterinary and resource-constrained settings. In veterinary practice, delays in diagnosis or prolonged turnaround times can lead to suboptimal treatment strategies and poorer clinical outcomes [4]. Therefore, there is a particularly urgent demand for rapid, reliable and cost-effective diagnostic tools to enable timely confirmation of fungal infections and to avoid unnecessary antifungal treatment [13,14].

Fungal cell walls are predominantly composed of glucans, which are essential for maintaining their structural integrity and functional diversity [15,16]. Among these glucans, β-1,3-glucan is the most abundant, typically accounting for 65% to 90% of the total glucan content in the cell wall [15]. Most fungal β-glucans consist primarily of β-1,3-linked glucose units, with varying amounts of β-1,6-linked branches. The abundance of β-1,6-glucan in fungal cell walls varies, but it is typically less abundant than β-1,3-glucan [15].

Given the differing abundance of these glucans, combining the detection of both β-1,3- and β-1,6-glucans may enhance the sensitivity and specificity of diagnostic assays. Such an approach may broaden the detection coverage, potentially making it more suitable for identifying fungal species with varying cell wall compositions. Simultaneously detecting both β-1,3-glucan and β-1,6-glucan may offer a more comprehensive diagnostic strategy, increasing the reliability and early detection of fungal infections, particularly when fungal load is low during the early stages of infection.

Currently, commercial diagnostic methods for detecting β-1,3-glucan predominantly rely on immunological principles, such as antibody-based or antigen-based detection of (1 → 3) β-D-glucan released into the bloodstream during invasive fungal infections [17,18,19]. Immunological methods for detecting β-1,3-glucan are associated with issues such as false positives, cross-reactivity, and limitations related to fungal species, as well as interference from variations in cell wall compositions across different fungi.

**Figure 1:**
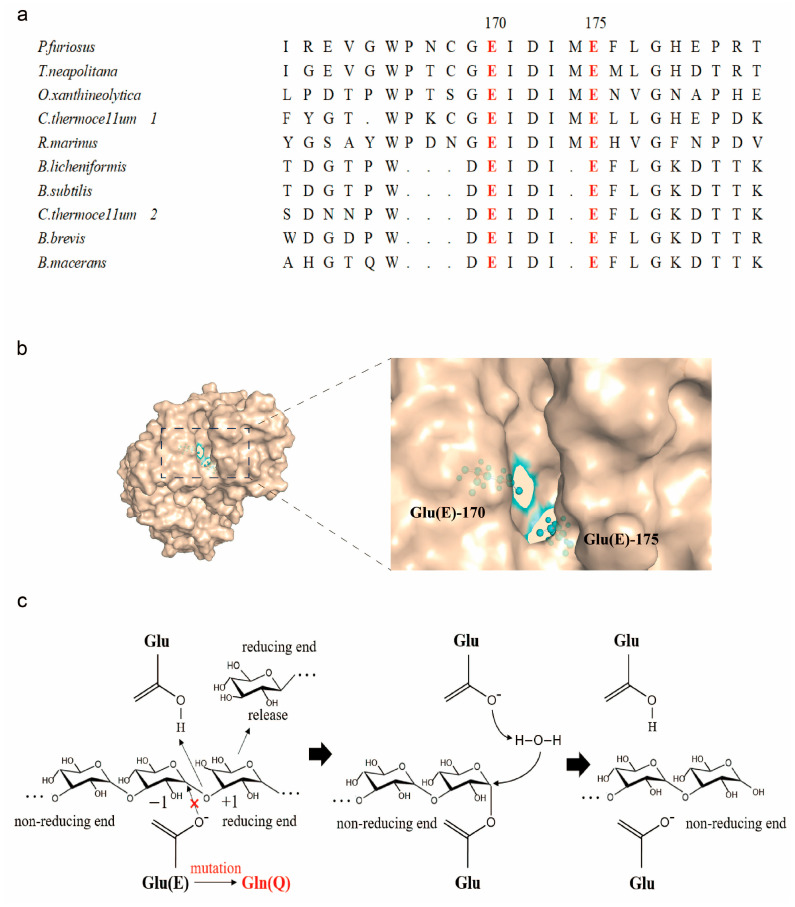
Catalytic Mechanism and Active Site Residues of β-1,3-Glucanase LamA from *Pyrococcus furiosus*. (**a**) Alignment of *Pyrococcus furiosus* LamA with other family 16 glycoside hydrolases, highlighting conserved glutamate residues (in red), which are identified as acid-base catalysts and active site nucleophiles. The sequences shown from top to bottom are *Pyrococcus furiosus* LamA; *Thermotoga neapolitana* laminarase; *Oerskovia xanthineolytica* β-1,3-glucanase; *Clostridium thermocellum* β-1,3(4)-glucanase LicA; *Rhodothermus marinus* β-glucanase; *Bacillus licheniformis* β-1,3-1,4-glucanase; *Bacillus subtilis* β-glucanase; *Clostridium thermocellum* β-1,3-1,4-glucanase LicB; *Bacillus brevis* β-1,3-1,4-glucanase; and *Bacillus macerans* β-1,3-1,4-glucanase. Residue positions are numbered according to the *P. furiosus* LamA sequence. (**b**) The catalytic residues of β-1,3-glucanase LamA from *Pyrococcus furiosus*. (**c**) The enzymatic reaction mechanism of β-1,3-glucanase LamA from *Pyrococcus furiosus* and a hypothesis on how mutations in catalytic residues (from Glu-E to Gln-Q) alter enzymatic activity, preserving binding ability but losing hydrolytic capability.

As an alternative to conventional β-1,3-glucan assays, we developed a glycoside hydrolase-based approach for β-1,3-glucan detection. Glycoside hydrolases catalyze hydrolysis via a double-displacement mechanism, where two glutamate residues act as the nucleophile and proton donor. The first glutamate attacks the glycosidic bond, forming a covalent intermediate, while the second stabilizes the structure by accepting a proton. After cleavage, water displaces the enzyme-substrate bond, completing the hydrolysis [20,21,22]. Mutations in catalytic residues can impair hydrolytic activity while preserving binding, as shown in GH30 endo-β-1,6-glucanase (Neg1), where Glu-225 and Glu-321 mutations eliminate hydrolysis but maintain glucan binding [23]. In LamA (GH16), sequence alignment identifies two conserved glutamates, Glu-170 and Glu-175 (Figure 1a), as the acid-base catalyst and nucleophile [21]. We hypothesize that mutating these residues will eliminate hydrolysis while preserving binding.

Based on this background, we chose the GH16 β-1,3-glucanase LamA from *Pyrococcus furiosus* in our study, targeting its catalytic residues Glu-170 and Glu-175 (Figure 1a). These glutamic acid residues were mutated to glutamine (Gln) to abolish the hydrolytic activity of LamA while maintaining its glucan-binding affinity. Meanwhile, we conducted a combined detection assay using the previously published *Neurospora*-derived endo-β-1,6-glucanase (Neg1) mutant, Neg1_E321Q_ [23]. This approach enables the creation of highly specific probes for detecting fungal β-1,3- and β-1,6-glucans in the sandwich ELISA format. The assay was further optimized and evaluated using reference fungal strains, simulated biological matrices, and preliminary clinical skin swab samples to assess its feasibility for broad-spectrum fungal screening.

Accordingly, the objective of this study was to develop a rapid, cost-effective, and broad-spectrum sandwich ELISA for detecting fungal β-1,3- and β-1,6-glucan signals using two catalytically inactive glucanase probes. Using pre-coated plates, the assay can be completed within approximately 40 min, providing a rapid and reliable approach for detecting a wide range of fungal species. This method is designed as a practical screening tool to indicate the presence of a meaningful fungal burden in suspected clinical samples, offering timely preliminary evidence of fungal involvement. While not intended for species-level identification, it can complement conventional diagnostic approaches, including clinical assessment, microscopy, fungal culture, PCR-based identification, and antifungal susceptibility testing. With further optimization and larger-scale clinical validation, this assay may support the future development of rapid fungal screening tools for veterinary diagnostics and potentially broader clinical applications.

## 2. Materials and Methods

### 2.1. Fungal Strains and Sample Preparation

Standard strains used in this study included laboratory-maintained reference strains of *Saccharomyces cerevisiae* and *Candida albicans*. Additionally, three dermatophyte species-*Microsporum canis*, *Trichophyton mentagrophytes*, and *Nannizzia gypsea* (formerly *Microsporum gypseum*)-and one dematiaceous mold, *Alternaria* sp., were isolated from veterinary cases and stored as glycerol stocks at −80 °C. Frozen fungal strains were streaked on YPD agar plates and incubated to revive colonies. For *Candida albicans*, plates were incubated at 37 °C, while other fungal strains were incubated at 30 °C. Single colonies were selected and inoculated into YPD broth under aerobic conditions. Cultures were used for subsequent experiments after reaching the desired growth stage.

For initial ELISA testing, all fungal strains, including *Saccharomyces cerevisiae*, *Candida albicans*, dermatophytes, and *Alternaria* sp., were suspended in phosphate-buffered saline (PBS). For further evaluation under more physiologically relevant conditions, *Candida albicans* cells were also suspended in RPMI-1640 medium containing 10% fetal bovine serum (FBS), simulating a more complex biological matrix. To mimic the dynamic in-blood survival state, *Candida albicans* cells (approximately 10^5^ cells/mL) were inoculated into RPMI-1640 medium supplemented with 10% FBS and incubated statically at 37 °C for 24 h. After incubation, supernatants were collected by centrifugation (1800× *g*, 10 min, 4 °C) and stored at −20 °C until further analysis [23].

### 2.2. Preparation of Fungal Cell-Wall β-Glucan Extracts

**Figure 2:**
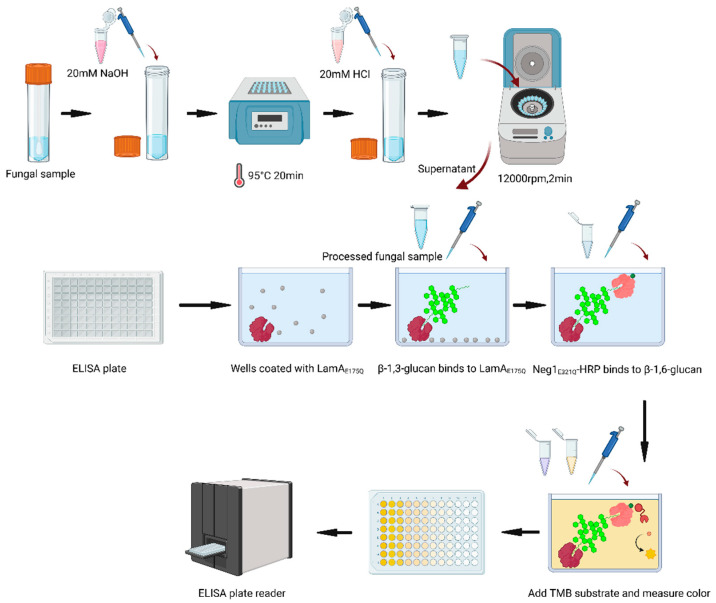
Fungal sample processing and sandwich ELISA for dual-glucan detection (LamA_E175Q_ as capture and HRP-conjugated Neg1_E321Q_ as detection).

Fungal cells or mycelial masses were harvested, washed once in phosphate-buffered saline (PBS, pH 7.4), and resuspended in PBS. Alkaline heat extraction was performed by adding NaOH to a final concentration of 20 mM and incubating the suspension at 95 °C for 20 min. Samples were centrifuged at 12,000× *g* for 2 min, and supernatants were immediately neutralized with 20 mM HCl (final pH 7.0–7.4). Neutralized extracts were aliquoted to minimize freeze–thaw cycles and stored at −20 °C until ELISA (Figure 2).

### 2.3. Construction and Expression of Mutant β-Glucanase

To generate inactive β-1,3-glucanase probes for β-1,3-glucan recognition, site-directed mutations were introduced into the catalytic residues of LamA from *Pyrococcus furiosus*. Glutamate residues at positions 170 and 175 were substituted with glutamine to generate LamA_E170Q_ and LamA_E175Q_, respectively. The mutations were introduced using a two-fragment PCR-based site-directed mutagenesis strategy, with reference to the method described by Yang et al. [24] and related *E. coli*-mediated homologous assembly methods [25,26]. For each mutation, the plasmid was divided into two PCR fragments with homologous overlapping ends. One overlap contained the intended mutation site, whereas the other overlap was located within the kanamycin-resistance gene. To construct LamA_E170Q_, two high-fidelity PCR reactions were performed in 25 μL reaction mixtures using pET-28a-pfLam-HS, a laboratory-constructed expression plasmid encoding His- and Strep-tagged pfLam, as the template. The His tag was used for affinity purification of the recombinant protein, whereas the Strep tag was used for tag-based detection in the indirect ELISA assays. The primer pairs LamA_E170Q_F (5′-GAACTGCGGCCAAATCGATATCATGGAATTCCTGGGTC-3′)/KanR (5′-CGGTTGCATTCGATTCCTGTTT-3′) and LamA_E170Q_R (5′-GACCCAGGAATTCCATGATATCGATTTGGCCGCAGTTC-3′)/KanF (5′-AAACAGGAATCGAATGCAACCG-3′) were used to generate two overlapping PCR fragments. To construct LamA_E175Q_, pET-28a-pfLam-HS was used as the template, and the primer pairs LamA_E175Q_F (5′-GAACTGCGGCGAAATCGATATCATGCAATTCCTGGGTC-3′)/KanR and LamA_E175Q_R (5′-GACCCAGGAATTGCATGATATCGATTTCGCCGCAGTTC-3′)/KanF were used in two separate 25 μL high-fidelity PCR reactions. A small aliquot of each PCR product was analyzed by 1% agarose gel electrophoresis to confirm the presence of the expected bands.

The remaining PCR products were treated with 0.5 μL DpnI at 37 °C for 30 min to remove the methylated parental plasmid template. After DpnI digestion, 2.5 μL of each of the two corresponding PCR products was mixed and transformed into 50 μL of chemically competent *E. coli* DH5α cells for plasmid recovery. The two PCR fragments were not intended to be maintained as linear DNA molecules; rather, successful in vivo assembly reconstructed a circular plasmid containing an intact origin of replication and a functional kanamycin-resistance gene. Transformants were selected on LB agar plates containing kanamycin. Positive colonies were cultured, and plasmids were extracted and subjected to Sanger sequencing by Sangon Biotech (Shanghai, China) to confirm the intended mutation and the correctness of the relevant reconstructed regions. Only sequence-verified recombinant plasmids were subsequently transformed into *E. coli* BL21 (DE3) cells for protein expression. The recombinant strains were cultured in LB medium containing kanamycin (50 μg/mL) and induced with 1 mM IPTG at 18 °C for 16 h. Cells were harvested by centrifugation and resuspended in Buffer A (5 mM imidazole, 50 mM Tris, 500 mM NaCl, pH 7.5). The cell suspension was disrupted using a JN-02C high-pressure cell disrupter at 1.0 MPa for three passes. The lysate was centrifuged at 12,000 rpm for 90 min, and the supernatant was filtered through a 0.45 μm membrane.

The filtered supernatant was loaded onto a HisTrap HP column using a peristaltic pump. After sample loading, the column was connected to an ÄKTA Pure protein purification system (Cytiva, Marlborough, MA, USA). The system was equilibrated with Buffer A at a flow rate of 1 mL/min, with the pressure alarm set to 0.5 MPa. The column was washed with Buffer A until the OD280 signal approached the baseline, followed by an additional wash with five column volumes of Buffer A. Bound proteins were eluted using different gradients of Buffer B (500 mM imidazole, 50 mM Tris, 500 mM NaCl, pH 7.5), and all elution fractions were collected. The column was subsequently washed with 100% Buffer B and then with five column volumes of ddH_2_O before storage.

Eluted fractions were analyzed by SDS-PAGE. Fractions containing the target proteins were pooled and concentrated using 30 kDa molecular weight cut-off ultrafiltration tubes to a final volume of approximately 2 mL. Protein concentrations were determined using the BCA assay, and the purified proteins were stored at −80 °C until further use.

### 2.4. Enzymatic Hydrolytic Activity Assay

The hydrolytic activity of wild-type and mutant enzymes was evaluated using laminarin (β-1,3-glucan, 1.0 mg/mL) as a model substrate. Reactions were carried out at a final enzyme concentration of 0.1 mg/mL in 50 mM phosphate buffer (pH 6.4) for LamA variants. Assays were incubated at the indicated temperatures, and aliquots were withdrawn at 0, 5, 10, 15, 30, 45, and 60 min. Each aliquot was immediately mixed with 100 µL of 3,5-dinitrosalicylic acid (DNS) reagent to terminate the reaction and allow color development. The release of reducing sugars was quantified by measuring absorbance at 540 nm using a microplate reader. All assays were performed in triplicate to ensure reproducibility.

### 2.5. ELISA Procedures

#### 2.5.1. Indirect ELISA for the Glucan Binding Affinities

High-binding 96-well plates were coated overnight at 4 °C with 100 µL per well of separately prepared two-fold serial dilutions of laminarin and pustulan, ranging from 10 to 0.15625 µg/mL and from 4 to 0.0625 µg/mL, respectively. After washing three times with PBST (PBS, 0.05% Tween-20), the plates were blocked with 1% BSA in PBST at 37 °C for 1 h. Mutant proteins LamA_E175Q_, LamA_E170Q_, and Neg1_E321Q_ were diluted in PBST containing 1% BSA and added to the wells (100 µL per well) with different concentrations of Laminarin for β-1,3-glucan binding and Pustulan for β-1,6-glucan binding, followed by incubation at 37 °C for 30 min. After washing, HRP-conjugated anti-Strep antibody (1:5000 in 1% BSA-PBST) was added and incubated at room temperature for 30 min. The reaction was developed with TMB substrate for 5 min and stopped with 50 µL of 1 M sulfuric acid. Absorbance was measured at 450 nm using a microplate reader, with triplicate measurements to ensure reproducibility.

#### 2.5.2. Indirect ELISA for Glucan Binding Specificity

*Saccharomyces cerevisiae* strains were cultured in 1 L YPD broth to mid-log phase and harvested by centrifugation (5000× *g*, 10 min). Cells were washed three times with ddH_2_O. For cell wall (CW) preparation, washed cell pellets were subjected to hot water extraction (~100 °C for 1 h) to remove soluble components, followed by centrifugation and washing with ddH_2_O. The CW pellets were then treated with an alkaline solution at 65 °C for 1 h with intermittent mixing to obtain the alkaline-insoluble fraction (AI). AI pellets were washed three times with ddH_2_O, lyophilized, weighed, and stored at room temperature.

To generate β-1,6-glucan-enriched AI-LamA supernatant, AI (10 mg) was incubated with 10 mL LamA (0.1 mg/mL in 50 mM phosphate buffer, pH 6.4) at 37 °C for 24 h. LamA alone was processed in parallel as a control. After centrifugation, supernatants were pooled and additional LamA (0.1 mg/mL) was added for 2 h at 37 °C to remove residual β-1,3-glucan. The pooled supernatant was then filtered through a 3 kDa ultrafiltration unit to remove LamA protein while retaining soluble β-1,6-glucan, followed by buffer exchange into ddH_2_O. The β-1,6-glucan content of the AI-LamA supernatant was estimated using Neg1_E321Q_ indirect ELISA with Pustulan as a reference. Supernatants were used immediately due to limited stability.

For indirect ELISA, high-binding 96-well polystyrene plates were coated overnight at 4 °C with 100 µL per well of the following polysaccharides (1 mg/mL each): laminarin (β-1,3-glucan), pustulan (β-1,6-glucan), mannan, CW, AI, and AI-LamA supernatant. Plates were washed three times with PBST (PBS containing 0.05% Tween-20) and blocked with 1% BSA in PBST at 37 °C for 1 h. Mutant proteins LamA_E175Q_ (5 µg/mL) and Neg1_E321Q_ (1 µg/mL) were prepared in PBST containing 1% BSA and added to the wells (100 µL per well) for 30 min at 37 °C. After washing, HRP-conjugated anti-Strep antibody (1:5000 in blocking buffer) was added for 30 min. Color development was performed using TMB substrate and stopped with 1 M H_2_SO_4_. Absorbance was measured at 450 nm. All measurements were performed in triplicate.

This method integrates the preparation of glucan-rich yeast cell wall fractions with specific glucanase-based probes, providing a reliable tool for the qualitative assessment of β-1,3- and β-1,6-glucans in yeast-derived samples.

#### 2.5.3. Sandwich ELISA for Dual-Glucan Detection

Plates were coated with the LamA_E175Q_ capture probe diluted in carbonate buffer (pH 9.6; 100 µL/well) and incubated overnight at 4 °C. After coating, the plates were washed three times with PBST and blocked with 1% BSA in PBS for 1 h at room temperature. The plates were washed again before sample addition.

The tested samples included laboratory-maintained fungal strains, simulated serum-containing fungal cultures, and clinical skin swab samples from veterinary patients with suspected dermatophytosis or negative controls. Sample pretreatment was performed as described in Section 2.2. Pretreated sample supernatants were added to the LamA_E175Q_-coated wells (100 µL/well) and incubated for 1 h at 37 °C to allow binding to the capture probe. After incubation, the plates were washed to remove unbound material. Detection was performed using Neg1_E321Q_ directly conjugated to HRP [HRP-conjugated Neg1_E321Q_, 1 µg/mL in blocking buffer], which was added to each well and incubated for 30 min at 37 °C. After a final washing step, TMB substrate was added for color development. The reaction was stopped with 1 M H_2_SO_4_, and the absorbance was measured at 450 nm using a microplate reader.

Each sample reaction was performed in three replicate wells. The overall workflow of the assay is summarized in Figure 2.

### 2.6. Conventional ITS PCR for Saccharomyces cerevisiae and Candida albicans

To evaluate the sensitivity of the developed ELISA, conventional PCR targeting the internal transcribed spacer (ITS) region was performed for *Saccharomyces cerevisiae* and *Candida albicans*. Universal fungal ITS primers ITS1 (5′-TCCGTAGGTGAACCTGCGG-3′) and ITS4 (5′-TCCTCCGCTTATTGATATGC-3′) [27] were used. For colony PCR, single yeast colonies were suspended in 20 mM NaOH and incubated at 98 °C for 10 min, followed by centrifugation to collect the supernatant, which served as the template. PCR amplification consisted of an initial denaturation at 95 °C for 10 min (1 cycle), followed by 30 cycles of 95 °C for 30 s, 55 °C for 30 s, and 72 °C for 15 s, and a final extension at 72 °C for 5 min. Reactions were then held at 4 °C. PCR products were visualized on 1.5% agarose gels.

### 2.7. Fungal Cell Quantification

Fungal cell quantification was performed using serial dilution and colony-forming unit (CFU) assays. For *Saccharomyces cerevisiae* and *Candida albicans*, overnight liquid cultures in YPD broth were prepared, and appropriate serial dilutions were plated on YPD agar. CFUs were counted after incubation at 30 °C (2 days) and 37 °C (2 days), respectively. For dermatophytes (*Microsporum canis*, *Trichophyton mentagrophytes*, *Nannizzia gypsea*) and *Alternaria* sp., liquid cultures were grown in YPD broth for 5–7 days, followed by serial dilution and plating on YPD agar. CFUs were counted after incubation at 30 °C (5–7 days). Due to the filamentous growth and hyphal clumping of dermatophytes and *Alternaria* sp., CFU counts for these species are approximate and should be interpreted as indicative of fungal biomass rather than precise quantitative measurements.

### 2.8. Collection and Processing of Clinical Skin Swab Samples from Veterinary Cases

To assess the clinical applicability of the assay, skin swab samples were collected from veterinary patients presenting with suspected dermatophyte infection, along with negative control samples from patients without clinical signs suggestive of dermatophytosis. Each sample was tested in parallel by conventional methods (fungal culture and PCR-based DNA sequencing) and the sandwich ELISA. Swabs were taken from lesion sites, suspended in PBS, vortexed, and subjected to alkaline-heat treatment to extract fungal β-glucans.

### 2.9. Establishment of Positive Cutoff Values for Sandwich ELISA in Fungal Detection: Statistical Approaches for Clinical and Laboratory Samples

In this study, the definition of a positive result for the sandwich ELISA was determined based on different criteria depending on the sample size and the type of sample analyzed.

For the clinical dermatophyte samples, the positive cutoff value was established using the mean absorbance (OD) of negative control samples plus three times the standard deviation (mean + 3 × SD). This method was selected due to the relatively larger sample size available, which allowed for a more robust statistical determination of the cutoff [28,29]. This approach ensures that any sample exhibiting absorbance values exceeding this threshold would be considered positive for fungal β-glucan structures.

In contrast, for all other ELISA experiments, including those involving laboratory strains and other fungal species, the positive cutoff was defined as a P/N ratio greater than 2.1. This threshold was adopted due to the limited sample sizes in these assays, as smaller sample sizes do not permit the same statistical analysis with high precision. A P/N ratio exceeding 2.1 has been widely accepted in the field as the standard for defining a positive result, as it reliably differentiates positive from negative signals while minimizing the risk of false positives [30].

These established cutoff criteria provide a consistent and reproducible method for interpreting ELISA results, ensuring the reliability and validity of the diagnostic assays across different sample types and conditions.

### 2.10. Protein Structural Visualization

The three-dimensional structure of LamA was obtained from the Protein Data Bank (PDB ID: 2VY0) and visualized using PyMOL version 3.1.1. The protein surface was displayed to show the catalytic pocket, and the catalytic residues Glu170 and Glu175 were highlighted and labeled according to their positions in the LamA sequence. An enlarged view of the catalytic region was generated in PyMOL to illustrate the spatial location of the two conserved glutamate residues. The final figure was exported from PyMOL and arranged for presentation.

## 3. Results

### 3.1. Generation and Validation of Catalytically Inactive Glucanase Mutants

**Figure 3:**
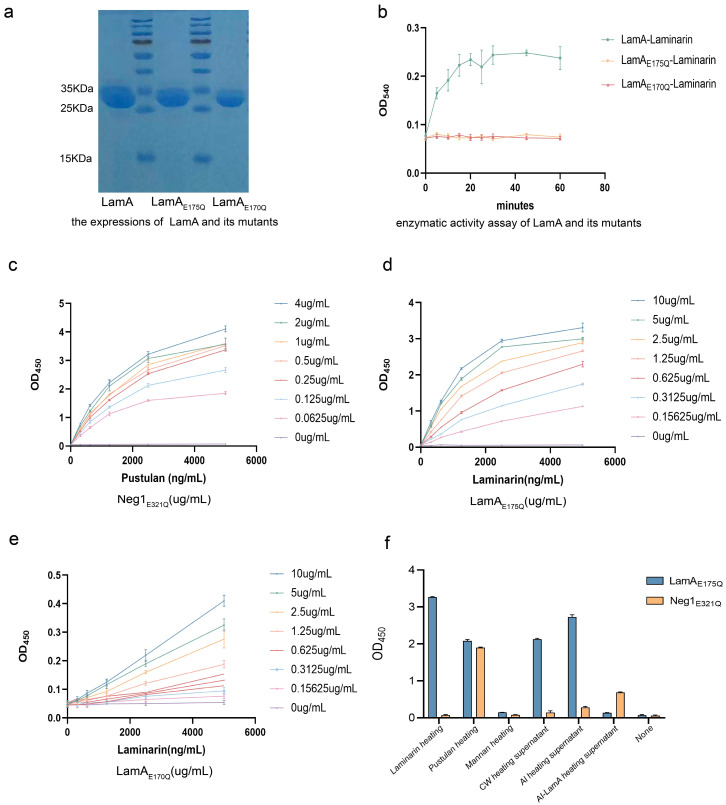
Overview of the characterization of LamA and its mutants, including protein expression, enzymatic activity, and detection specificity. (**a**) SDS-PAGE analysis of LamA, LamA_E175Q_ and LamA_E170Q_. (**b**) Enzymatic activity assay of LamA and its mutants. (**c**) Detection of pustulan using the HRP-conjugated Neg1_E321Q_ mutant, as reported in the previous literature. (**d**) Detection of Laminarin using LamA_E175Q_. (**e**) Detection of Laminarin using LamA_E170Q_. (**f**) Specificity of binding by LamA_E175Q_ (5 µg/mL) and Neg1_E321Q_ (1 µg/mL) (CW: Suspension, cell wall prepared from yeast cell walls; AI: Suspension, cell wall prepared from CW after alkali treatment; AI-LamA: The alkaline-insoluble (AI) fraction was treated with β-1,3-glucanase LamA to yield a cell wall β-1,6-glucan).

Based on the conserved catalytic mechanism of glycoside hydrolases, the key catalytic residues involved in substrate cleavage and stabilization were summarized schematically (Figure 1c). These residues provided the structural basis for selecting the corresponding mutation sites used in this study. Mutations in the catalytic residues can significantly alter hydrolytic activity. For instance, in the GH30 family endo-β-1,6-glucanase (Neg1), mutations at Glu-225 and Glu-321 to glutamine eliminated hydrolytic activity while preserving glucan binding [23]. In the case of *Pyrococcus furiosus* LamA (GH16), sequence alignment with other members of family 16 glycoside hydrolases reveals two highly conserved glutamate residues (Figure 1a): Glu-170 and Glu-175 [31], which have been identified as crucial for acid-base catalysis and nucleophilic attack (Figure 1b) [21]. Based on these findings, we hypothesize that mutating these residues could similarly eliminate hydrolytic activity (Figure 1c) while preserving binding affinity, as seen in Neg1. To test this hypothesis, we generated two site-directed mutants of the β-1,3-glucanase LamA (GH16 family) from *Pyrococcus furiosus*: LamA_E170Q_ and LamA_E175Q_. SDS-PAGE analysis confirmed the successful expression and purification of both wild-type and mutant proteins in *E. coli*, with the expected molecular mass of approximately 30 kDa (Figure 3a). To evaluate enzymatic activity, a DNS reducing sugar assay was performed using laminarin (β-1,3-glucan) as a substrate. As anticipated, the wild-type LamA exhibited robust hydrolytic activity, efficiently breaking down laminarin. In contrast, the mutants LamA_E170Q_ and LamA_E175Q_ showed no measurable increase in reducing sugars, indicating a complete loss of catalytic activity (Figure 3b). These findings confirmed that the mutations effectively disabled the enzymatic function, validating these variants as catalytically inactive probes suitable for use in binding-based detection.

### 3.2. Glucan-Binding Capacity of Mutant Proteins

The binding properties of the mutant glucanases were evaluated using indirect ELISA with laminarin (β-1,3-glucan) and pustulan (β-1,6-glucan) as binders. Among the β-1,3-glucan-specific mutants, LamA_E175Q_ showed a stronger ELISA signal than LamA_E170Q_ for laminarin and was therefore selected for subsequent assay development (Figure 3d,e). For the β-1,6-glucan-specific probe, Neg1_E321Q_ exhibited strong and specific binding to pustulan (Figure 3c), consistent with the published data [23]. These results led to the selection of LamA_E175Q_ and Neg1_E321Q_ as the preferred probes for subsequent dual-probe sandwich ELISA development.

### 3.3. Specificity and Binding Profile of LamA_E175Q_ and Neg1_E321Q_ Towards β-1,3- and β-1,6-Glucans in Indirect ELISA

Indirect ELISA results demonstrated that LamA_E175Q_ (specific for β-1,3-glucan) did not bind to mannans, but it effectively bound to commercial β-1,3-glucan (laminarin). Although LamA_E175Q_ exhibited some binding to β-1,6-glucan (pustulan), further analysis involving AI (β-glucan-rich, including β-1,3-glucan and β-1,6-glucan) and AI-LamA (β-1,6-glucan) showed that LamA addition significantly reduced LamA_E175Q_’s signal for β-1,3-glucan. These findings confirm the specificity of LamA_E175Q_ for β-1,3-glucan. Although pustulan is commonly used as a β-1,6-glucan standard, natural pustulan-related preparations may contain β-1,3-glucan-associated components or structural heterogeneity. Pereyra et al. reported that polysaccharide fractions from *Lasallia pustulata* include pustulan as well as β-(1 → 3)-glucan-containing fractions. Therefore, the residual LamA_E175Q_ signal observed with pustulan is not unexpected (Figure 3f) [32].

For Neg1_E321Q_ (specific for β-1,6-glucan), binding to commercial β-1,6-glucan (pustulan) was confirmed. The addition of LamA resulted in a higher detected signal for β-1,6-glucan, consistent with previous studies demonstrating the high specificity of Neg1_E321Q_ for β-1,6-glucan [23] (Figure 3f). These results further demonstrate the nuanced interactions of these engineered probes with β-glucans, highlighting their potential for refined fungal diagnostics.

### 3.4. Optimization of Dual-Probe Sandwich ELISA

**Figure 4:**
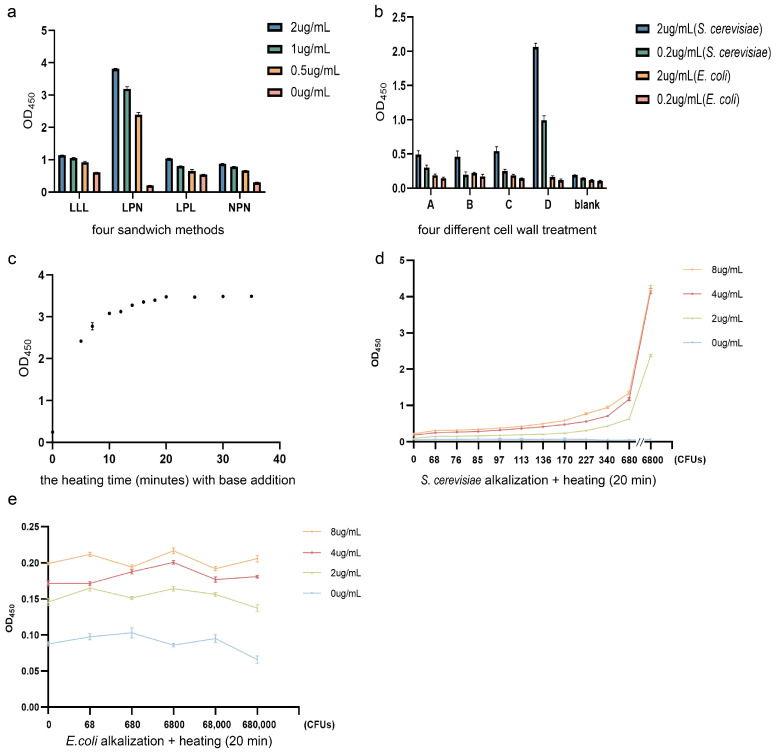
Overview of optimizing treatment conditions and detection methods for *Saccharomyces cerevisiae* and *E. coli* cell walls. (**a**) Comparisons of four different sandwich formats: LLL: LamA_E175Q_–Laminarin (1000 ng/mL)–HRP-conjugated LamA_E175Q_; LPN: LamA_E175Q_–Pustulan (1000 ng/mL)–HRP-conjugated Neg1_E321Q_; LPL: LamA_E175Q_–Pustulan (1000 ng/mL)–HRP-conjugated LamA_E175Q_; NPN: Neg1_E321Q_–Pustulan (1000 ng/mL)–HRP-conjugated Neg1_E321Q_. (**b**) Effect of four different cell wall treatment conditions on detection performance. A: No treatment; B: Heat at 95 °C for 5 min; C: Add acid (20 mM), then heat at 95 °C for 5 min; D: Add base (20 mM), then heat at 95 °C for 5 min. (**c**) Optimization of the heating time (5–35 min) with base addition for LamA_E175Q_–processed *Saccharomyces cerevisiae*–HRP-conjugated Neg1_E321Q_. (**d**) Effect of base treatment and heating for 20 min on the LamA_E175Q_–processed *Saccharomyces cerevisiae*–HRP-conjugated Neg1_E321Q_. Note: the final point (6800 CFUs) is 10-fold higher than the preceding point (680 CFUs). (**e**) Effect of base treatment and heating for 20 min on the LamA_E175Q_–processed *E. coli*–HRP-conjugated Neg1_E321Q_.

We evaluated multiple configurations for the sandwich ELISA, using pustulan as the β-glucan substrate. Among the tested formats, the combination of LamA_E175Q_ (capture)–pustulan–HRP-conjugated Neg1_E321Q_ (detection) yielded the highest OD_450_ values with minimal background noise, providing a higher signal-to-noise ratio (Figure 4a). In contrast, the Neg1_E321Q_ (capture)–HRP-conjugated Neg1_E321Q_ (detection) configuration demonstrated much lower performance at equivalent glucan concentrations. We further optimized the assay by systematically testing different concentrations of the LamA_E175Q_ capture probe, in combination with a fixed concentration of 1 µg/mL Neg1_E321Q_ for detection, identifying optimal conditions for maximal assay sensitivity (Figure 2).

### 3.5. Detection of Saccharomyces cerevisiae and Optimization of Cell-Wall Processing Conditions

To establish an effective sample-processing workflow, we compared various pretreatment conditions for *Saccharomyces cerevisiae* cells, including no treatment, acid treatment, and alkaline treatment. The alkaline treatment consistently produced the highest and most reproducible β-glucan signals (Figure 4b). We then optimized the heating duration within the alkaline protocol, testing 0 to 35 min at 95 °C. The OD_450_ response increased with heating time up to 20 min, at which point a reproducible maximum was reached (Figure 4c). Extending the incubation time beyond 20 min did not provide any additional benefit. The final protocol of 20 min alkaline heating at 95 °C was established as the standard processing condition (Figure 4d). The sandwich ELISA exhibited a dose-dependent response when *Saccharomyces cerevisiae* was suspended in PBS. Strong signals were observed at approximately 6.8 × 10^3^ CFUs/reaction (100 µL). The positive threshold, defined as a P/N ratio of 2.1, was reached at approximately 136 CFUs/reaction (100 µL). Specificity was confirmed by showing that *E. coli* subjected to the same alkaline-heating conditions remained negative, indicating that the optimized protocol did not cause nonspecific reactivity (Figure 4e). Under these optimized conditions, the sandwich ELISA displayed a clear, dose-dependent response to *Saccharomyces cerevisiae*.

### 3.6. Detection of Candida albicans

#### 3.6.1. Detection of *Candida albicans* in PBS

**Figure 5:**
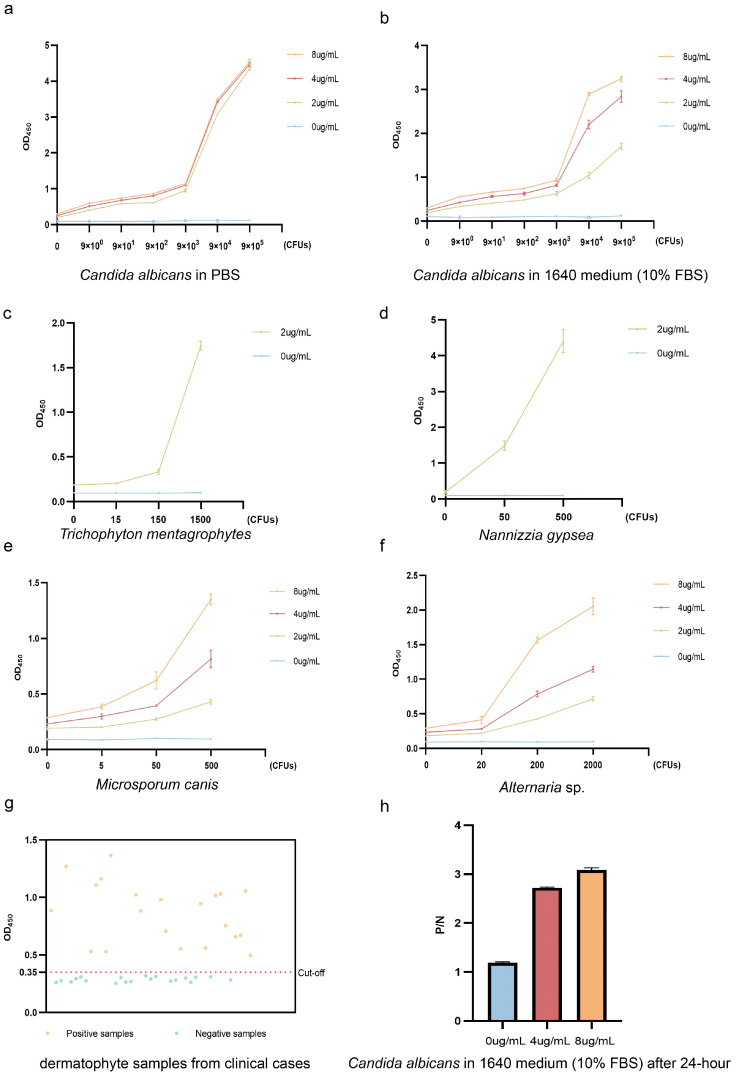
Evaluation of the LamA_E175Q_–sample–HRP-conjugated Neg1_E321Q_ assay for detecting *Candida albicans*, dermatophytes, and *Alternaria* sp. in various media and clinical samples. (**a**) Detection of laboratory-preserved *Candida albicans* in PBS using LamA_E175Q_ (2 µg/mL, 4 µg/mL and 8 µg/mL)–sample–HRP-conjugated Neg1_E321Q_. (**b**) Detection of laboratory-preserved *Candida albicans* in 1640 medium (10% FBS) using LamA_E175Q_ (2 µg/mL, 4 µg/mL and 8 µg/mL)–sample–HRP-conjugated Neg1_E321Q_. (**c**) Detection of laboratory-preserved *Trichophyton mentagrophytes* in PBS using LamA_E175Q_ (2 µg/mL)–sample–HRP-conjugated Neg1_E321Q_. (**d**) Detection of laboratory-preserved *Nannizzia gypsea* (*formerly Microsporum gypseum*) in PBS using LamA_E175Q_ (2 µg/mL)–sample–HRP-conjugated Neg1_E321Q_. (**e**) Detection of laboratory-preserved *Microsporum canis* in PBS using LamA_E175Q_ (2 µg/mL, 4 µg/mL and 8 µg/mL)–sample–HRP-conjugated Neg1_E321Q_. (**f**) Detection of laboratory-preserved *Alternaria* sp. in PBS using LamA_E175Q_ (2 µg/mL, 4 µg/mL and 8 µg/mL)–sample–HRP-conjugated Neg1_E321Q_. (**g**) Detection of dermatophytes in clinical samples using LamA_E175Q_ as the capture probe and HRP-conjugated Neg1_E321Q_ as the detection probe in PBS. When the concentration of LamA_E175Q_ is 8 μg/mL, the red dashed line represents the positive cutoff threshold (mean absorbance of negative control samples + 3 × standard deviation). (**h**) Detection of *Candida albicans* in 1640 medium (10% FBS) culture supernatant after 24 h incubation using LamA_E175Q_ (4 µg/mL and 8 µg/mL)–sample–HRP-conjugated Neg1_E321Q_.

The sandwich ELISA exhibited a dose-dependent response when *Candida albicans* was suspended in PBS. Strong signals were observed at approximately 9 × 10^5^ CFUs/reaction (100 µL). The positive threshold, defined as a P/N ratio of 2.1, was reached at approximately 90 CFUs/reaction (100 µL) (Figure 5a).

#### 3.6.2. Performance in RPMI-1640 with 10% FBS

The performance of the sandwich ELISA was further evaluated under biologically relevant conditions, with *Candida albicans* suspended in RPMI-1640 medium supplemented with 10% FBS. The assay exhibited dose–response characteristics comparable to those observed in PBS, with strong positive signals at approximately 9 × 10^5^ CFUs/reaction (100 µL). The positive threshold, defined as a P/N ratio of 2.1, was reached at approximately 90 CFUs/reaction (100 µL), suggesting that the assay retained comparable sensitivity in the presence of complex serum components (Figure 5b).

#### 3.6.3. Simulated In-Blood Conditions

To mimic serum-containing, blood-relevant conditions and assess the detectability of soluble fungal glucans released by *Candida albicans*, culture supernatants obtained after 24 h static incubation at 37 °C in RPMI-1640 supplemented with 10% FBS were analyzed using the LamA_E175Q_ (capture)–HRP-conjugated Neg1_E321Q_ (detection) dual-probe sandwich ELISA.

Clear signals were observed within the 8 µg/mL and 4 µg/mL LamA_E175Q_ coating range, with P/N ratios consistently exceeding 2.1 (Figure 5h).

These results demonstrate that the dual-probe sandwich ELISA enables sensitive detection of soluble, released fungal glucans from *Candida albicans* under serum-containing, blood-mimetic conditions.

### 3.7. Comparison of Our Method with Conventional ITS PCR for Saccharomyces cerevisiae and Candida albicans

We compared the sensitivity of our sandwich ELISA with conventional PCR, targeting the ITS region in the genomes of *Saccharomyces cerevisiae* and *Candida albicans*. For *Saccharomyces cerevisiae*, our sandwich ELISA, employing LamA_E175Q_ (β-1,3-glucan probe) for capture and Neg1_E321Q_ (β-1,6-glucan probe) for detection, demonstrated a detection threshold of approximately 136 CFUs per reaction, with a P/N value exceeding 2.1, whereas conventional PCR using commercially available universal fungal ITS primers exhibited a positive cutoff of approximately 612 CFUs per reaction (Supplementary Figure S1). For *Candida albicans*, the detection threshold of our sandwich ELISA was approximately 90 CFUs per reaction, compared to approximately 760 CFUs per reaction for conventional ITS PCR (Supplementary Figure S2). These results indicate that, under the experimental conditions of this study, our sandwich ELISA exhibited higher detection sensitivity than conventional PCR, particularly in detecting low-burden fungal infections.

### 3.8. Detection of Veterinary Dermatophytes

The sandwich ELISA was also used to detect dermatophytes, including *Microsporum canis*, *Trichophyton mentagrophytes*, and *Nannizzia gypsea*. *Trichophyton mentagrophytes* generated clear positive signals at approximately 1.5 × 10^3^ CFU-equivalent/reaction, while *Microsporum canis* and *Nannizzia gypsea* both showed clear positive signals at approximately 5 × 10^2^ CFU-equivalent/reaction (Figure 5c–e).

### 3.9. Detection of Alternaria sp.

Finally, we extended the sandwich ELISA to include *Alternaria* sp., a representative dematiaceous mold. The assay demonstrated sensitivity for *Alternaria* sp., with strong positive signals observed at approximately 2 × 10^3^ CFU-equivalent/reaction (Figure 5f), further showcasing the broad applicability of the ELISA across a variety of clinically relevant fungal pathogens.

### 3.10. Application to Clinical Skin Swab Samples from Veterinary Cases

The clinical applicability of the sandwich ELISA was assessed using skin swab samples collected from veterinary patients with suspected dermatophyte infection and negative control samples from patients without clinical signs suggestive of dermatophytosis. Each sample was tested in parallel by the sandwich ELISA and conventional fungal culture. Among the collected samples, 21 were confirmed positive, and 20 served as negative controls by conventional methods (fungal culture and PCR-based DNA sequencing). Using the optimized sandwich ELISA (LamA_E175Q_ as the capture probe and HRP-conjugated Neg1_E321Q_ as the detection probe) with a LamA_E175Q_ coating concentration of 8 μg/mL, the negative control range was determined from the 20 negative samples, and the positive threshold was set at 0.35. Applying this threshold, all 21 culture-positive samples produced detectable OD_450_ signals above the established cutoff, showing 100% concordance with traditional methods (fungal culture and PCR-based DNA sequencing) (Figure 5g, Table 1) and indicating the presence of fungal β-1,3/β-1,6-glucan structures in the clinical specimens.

This direct-from-swab detection demonstrates the feasibility of employing the sandwich ELISA for rapid, culture-free diagnosis of dermatophyte infections in veterinary clinical practice.

## 4. Discussion

Fungal infections impose substantial clinical and economic burdens in both human and veterinary medicine. Culture and microscopy are widely used but are often time-consuming, operator-dependent, and insufficiently sensitive in low-burden infections. Although ITS-based PCR improves sensitivity and coverage, it can be constrained by specialized instrumentation and personnel, limiting rapid and point-of-care deployment. Therefore, rapid, cost-effective, and practical methods with adequate sensitivity for low-level fungal detection remain needed. To address this gap, we developed a glycoside hydrolase-based sandwich immunoassay targeting β-1,3- and β-1,6-glucans, providing a streamlined, accessible, and cost-effective workflow to support timely detection and early clinical decision-making.

The sandwich ELISA demonstrated a detection threshold of 136 CFUs per reaction for *Saccharomyces cerevisiae*, which was lower than that of conventional ITS-based PCR. For *Candida albicans*, the detection threshold of the ELISA was also lower than that of conventional ITS-PCR. These results suggest that, under the experimental conditions of this study, the sandwich ELISA offered higher detection sensitivity than conventional ITS-based PCR for *Saccharomyces cerevisiae* and *Candida albicans*, supporting its potential as a complementary approach for fungal detection.

Furthermore, this assay demonstrated effective detection of dermatophytes and *Alternaria* sp., highlighting its broad applicability for detecting diverse fungal pathogens. In this preliminary clinical cohort, the sandwich ELISA identified β-glucan signals in all 21 culture-positive dermatophyte samples, while no signal was detected in 20 negative samples, suggesting the potential clinical applicability of this approach. Dermatophytes are among the most common fungal pathogens in companion animals, and they also pose a zoonotic risk to humans. The excellent performance in clinical samples highlights the practical applicability of our ELISA in real-world diagnostics, where speed and accuracy are crucial for effective infection management.

The sandwich ELISA also performed well in blood-mimetic systems for *Candida albicans*, such as RPMI-1640 with 10% fetal bovine serum. In addition, soluble β-1,3- and β-1,6-glucans released into the supernatant of *Candida albicans* cultures following centrifugation could be detected, demonstrating the capability of the assay to detect specific fungal glucans in complex serum-containing matrices. This robustness is especially important for diagnosing systemic fungal infections, where early detection is critical to prevent severe complications [1,33,34]. As blood sample collection is subject to ethical and biosafety considerations, future studies will aim to expand testing to include blood samples from both humans and animals under appropriate conditions. Our method is designed for broad-spectrum fungal detection and is capable of identifying various fungal species. However, its specificity for diagnosing a particular fungal infection still requires further refinement. Additionally, using pre-coated plates, the ELISA workflow can be completed within approximately 40 min, providing a rapid assay, although further optimization may be required to enhance sensitivity, particularly for low-burden infections.

The sandwich ELISA developed in this study is conceptually related to the β-1,6-glucan detection system reported by Yamanaka et al. [23], in which the catalytically inactive β-1,6-glucanase mutant Neg1_E321Q_ was used as a β-1,6-glucan-specific recognition probe. In contrast, our assay uses LamA_E175Q_ as the capture probe and chemically HRP-conjugated His-tagged Neg1_E321Q_ as the detection probe, establishing a heterologous dual-probe format for simultaneous β-1,3- and β-1,6-glucan recognition. Under our experimental conditions, this dual-probe configuration produced stronger and more consistent signals than using Neg1_E321Q_-based homologous probe configurations and was therefore selected for further assay development. This comparison is limited to the configurations tested in this study. Further studies are needed to evaluate analytical specificity, clinical applicability, and performance across different sample types. Although β-glucans are typical fungal cell-wall components, β-glucan-like polysaccharides are not completely exclusive to fungi. For example, β-(1 → 3),β-(1 → 6)-linked glucans have been reported in certain rhizobial or plant-associated environmental bacteria, such as *Bradyrhizobium japonicum* [35]. However, these bacterial glucans are mainly osmoregulated periplasmic cyclic glucans, rather than abundant exposed cell-wall structural components comparable to fungal β-glucans. *Bradyrhizobium japonicum* is also not a common animal skin pathogen or a major bacterial agent in routine veterinary dermatological infections. Therefore, while cross-reactivity with such special bacterial glucans cannot be excluded without direct testing, their clinical relevance as routine interfering organisms is likely limited. Future studies should include a broader bacterial panel, including common skin, wound, and systemic bacterial pathogens, as well as bacterial-fungal mixed samples, to further evaluate assay specificity.

Environmental fungi and commensal yeasts may be present on animal skin, hair, or sampling surfaces, but under the current assay conditions, trace-level contamination did not generate positive signals. In 20 negative veterinary samples, all remained negative, suggesting common surface fungi did not affect assay readout. Thus, a positive result indicates a meaningful fungal glucan burden, warranting further clinical evaluation. Consistent with the general use of β-D-glucan as an adjunctive biomarker [19], this assay is intended as a rapid, low-cost, broad-spectrum screening tool, particularly when the etiological agent is unknown or species-specific PCR may fail. Positive results should be interpreted alongside clinical signs, sampling site, microscopy, culture, PCR, and, when necessary, antifungal susceptibility testing for definitive diagnosis.

In conclusion, the sandwich ELISA developed in this study provides a rapid, cost-effective, and complementary approach for detecting fungal β-1,3- and β-1,6-glucan signals. By combining β-1,3- and β-1,6-glucan recognition, this assay may improve the detection coverage of fungal glucan signals and serve as a practical screening tool for suspected fungal samples. However, it is not intended for species-level identification and should be interpreted together with clinical findings and conventional diagnostic methods. Further validation using larger clinical cohorts, broader fungal panels, and different sample types will be needed to fully evaluate its diagnostic performance and clinical applicability.

## 5. Conclusions

In this study, we developed a sandwich ELISA to detect β-1,3- and β-1,6-glucans, essential components of the fungal cell wall, using catalytically inactive glucanase-derived probes. The assay enables dual glucan recognition, providing broader detection coverage across diverse fungal species. Under the experimental conditions, it demonstrated higher sensitivity than conventional ITS-based PCR for *Saccharomyces cerevisiae* and *Candida albicans* and successfully detected dermatophytes and *Alternaria* sp., including in blood-mimetic media. In a preliminary clinical cohort, the assay identified β-glucan signals in all 21 culture-positive dermatophyte samples and showed negative results in 20 negative samples, supporting its utility as a broad-spectrum screening tool. While not intended for species-level identification, this method can complement conventional diagnostic approaches, and further validation using larger clinical cohorts and a wider range of fungal species will be important to fully evaluate its applicability and robustness.

## Figures and Tables

**Table 1:** The comparison of clinical sample detection results between traditional methods (fungal culture and PCR-based DNA sequencing) and the sandwich ELISA method we developed (The rows indicate the results obtained using the sandwich ELISA developed in this study, whereas the columns indicate the results obtained using the traditional methods. The symbol “+” indicates a positive result, “−” indicates a negative result, and “Total” indicates the total number of samples in each corresponding category).

		Fungal Culture and PCR-Based DNA Sequencing		
		+	−	Total
Sandwich ELISA (this study)	+	21	0	21
	−	0	20	20
	Total	21	20	41

## Data Availability

Data will be made available on request.

## Supplementary Materials

The following supporting information can be downloaded at https://www.mdpi.com/article/10.3390/jof12060448/s1: Figure S1: Detection of *Saccharomyces cerevisiae* using conventional ITS-based PCR; Figure S2: Detection of *Candida albicans* using conventional ITS-based PCR.
