# Supplementary material for: Development of an Immunoassay Platform Targeting β-1,3- and β-1,6-Glucans for Rapid Detection of Fungi
Source: J Fungi (Basel). 2026 Jun 19;12(6):448. doi: 10.3390/jof12060448 (PMC13301891; doi:10.3390/jof12060448)
Supplement: Supplementary file 1 [file jof-12-00448-s001.zip › jof-4326605-supplementary materials.pdf]

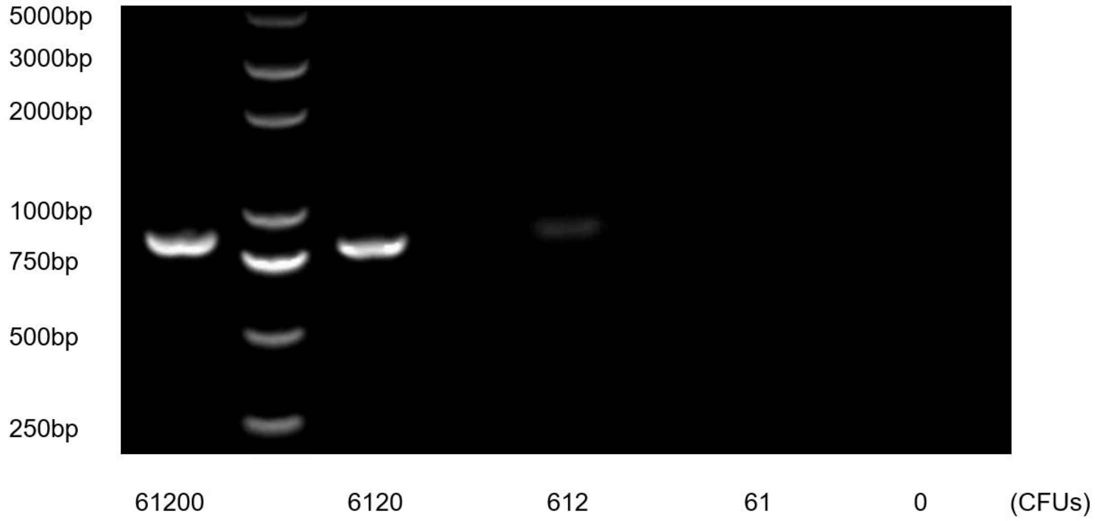

Figure S1. Detection of *Saccharomyces cerevisiae* using conventional ITS-based PCR.

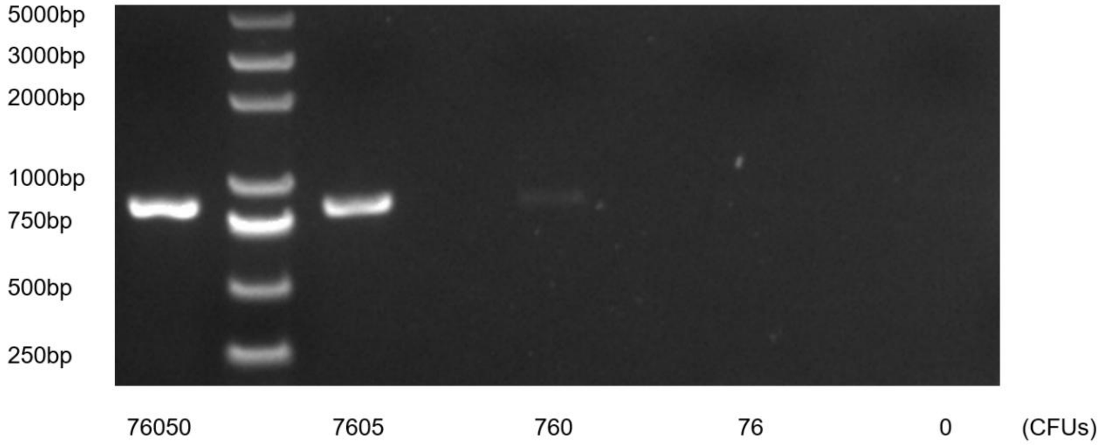

Figure S2. Detection of *Candida albicans* using conventional ITS-based PCR.
